# Antiviral Resistance and Correlates of Virologic Failure in the first Cohort of HIV-Infected Children Gaining Access to Structured Antiretroviral Therapy in Lima, Peru: A Cross-Sectional Analysis

**DOI:** 10.1186/1471-2334-13-1

**Published:** 2013-01-02

**Authors:** Barbara A Rath, Max von Kleist, Maria E Castillo, Lenka Kolevic, Patricia Caballero, Giselle Soto-Castellares, Angela M Amedee, James E Robinson, David K Katzenstein, Russell B Van Dyke, Richard A Oberhelman

**Affiliations:** 1 Department of Pediatrics, Division of Pneumonology-Immunology, Charité University Medical Center, Berlin, Germany; 2 Department of Pediatrics, Division of Infectious Diseases, Tulane University Health Sciences Center, New Orleans, Louisiana, USA; 3 Department of Mathematics and Computer Science, Free University Berlin, Berlin, Germany; 4 Infectious Diseases Service, Instituto Nacional de Salud del Niño Lima, Peru; 5 Executive Directorate of Research, National Institute of Health, Lima, Peru; 6 Asociación Benéfica PRISMA, Lima, Peru; 7 Department of Microbiology, Immunology & Parasitology, Louisiana State University Health Sciences Center, New Orleans, Louisiana, USA; 8 Center for AIDS Research, Stanford University, Stanford, Palo Alto, USA; 9 Department of Pediatrics, Universidad Peruana Cayetano Heredia, Lima, Peru

## Abstract

**Background:**

The impact of extended use of ART in developing countries has been enormous. A thorough understanding of all factors contributing to the success of antiretroviral therapy is required. The current study aims to investigate the value of cross-sectional drug resistance monitoring using DNA and RNA oligonucleotide ligation assays (OLA) in treatment cohorts in low-resource settings. The study was conducted in the first cohort of children gaining access to structured ART in Peru.

**Methods:**

Between 2002–5, 46 eligible children started the standard regimen of AZT, 3TC and NFV Patients had a median age of 5.6 years (range: 0.7-14y), a median viral load of 1.7·10^5^ RNA/ml (range: 2.1·10^3^ – 1.2·10^6^), and a median CD4-count of 232 cells/μL (range: 1–1591). Of these, 20 patients were classified as CDC clinical category C and 31/46 as CDC immune category 3. At the time of cross-sectional analysis in 2005, adherence questionnaires were administered. DNA OLAs and RNA OLAs were performed from frozen PBMC and plasma, RNA genotyping from dried blood spots.

**Results:**

During the first year of ART, 44% of children experienced virologic failure, with an additional 9% failing by the end of the second year. Virologic failure was significantly associated with the number of resistance mutations detected by DNA-OLA (p < 0.001) during cross-sectional analysis, but also with low immunologic CDC-scores at baseline (p < 0.001). Children who had been exposed to unsupervised short-term antiretrovirals before starting structured ART showed significantly higher numbers of resistance mutations by DNA-OLA (p = 0.01). Detection of M184V (3TC resistance) by RNA-OLA and DNA-OLA demonstrated a sensitivity of 0.93 and 0.86 and specificity of 0.67 and 0.7, respectively, for the identification of virologic failure. The RT mutations N88D and L90M (NFV resistance) detected by DNA-OLA correlated with virologic failure, whereas mutations at RT position 215 (AZT resistance) were not associated with virologic failure.

**Conclusions:**

Advanced immunosuppression at baseline and previous exposures to unsupervised brief cycles of ART significantly impaired treatment outcomes at a time when structured ART was finally introduced in his cohort. Brief maternal exposures to with AZT +/− NVP for the prevention of mother-to-child transmission did not affect treatment outcomes in this group of children. DNA-OLA from frozen PBMC provided a highly specific tool to detect archived drug resistance. RNA consensus genotyping from dried blood spots and RNA-OLA from plasma consistently detected drug resistance mutations, but merely in association with virologic failure.

## Background

Antiretroviral therapy (ART) has, for the past years, increased the hope for survival of millions of people living with the human immunodeficiency virus (HIV) worldwide, adults as well as children. A clear survival advantage was achieved for HIV-infected patients with a dramatic decrease in new AIDS cases [1]. Immune reconstitution ensues when viral replication can be suppressed successfully over time [2].

Once a first-line regimen has failed however, the reasons for such failure may be complex, including malnutrition and co-morbidities leading to poor absorption of medications. Lack of economic resources and education may further complicate the already difficult adherence to complex medication schedules [3-11]. Some patients may have been pre-exposed to intermittent or erratic courses of antiretrovirals through aid programs, private activities and contacts abroad. HIV-infected children may have also been infected with a resistant maternal virus through mother-to-child transmission (MTCT) [12,13]. In resource-limited settings where medications for standard first-line ART medications are often purchased *en bloc* and large groups of patients are started on ART simultaneously, cross-sectional drug resistance testing may be particularly useful.

This study aims to test the value and feasibility of cross-sectional resistance testing as well as innovative tools to display disease progression or clinical/immunological improvement in the first cohort of children starting ART in Peru. With Global Fund support, structured ART first became available in August 2002 to a select group of HIV-infected children at the Instituto Nacional de Salud del Niño (INSN) in Lima, based on the criteria established by the Guideline for the Management of the HIV-Infected Child by the Peruvian Ministry of Health (MINSA) [14-17].

In contrast to a neonatal cohort starting ART several years later, the majority of patients in this first cohort at the INSN were school-age, had already progressed to AIDS when starting ART and were born before the broad introduction of prevention of mother-to-child transmission (pMTCT) programs in Peru [18]. Therefore, most patients were considered ART-naive prior to starting the Peruvian standard first-line regimen, consisting azidothymidine (AZT, 100 mg/m^2^ every 12 hours) with lamivudine (3TC, 4 mg/Kg. every 12 hours) and nelfinavir (NFV, 25 mg/Kg. every 8 hours) [17].

At the time of introduction of ART in Peru, access to drug resistance testing was still limited. To save cost, alternative testing methodologies and transportation modalities were sought, such as the Oligonucleotide Ligation Assay (OLA) [19-21] and filter cards for the transportation of blood samples as dried spots [22-26].

The aims of the study were:

1. To determine the prevalence of antiretroviral drug resistance in children with virologic failure versus no virologic failure.

2. To evaluate the sensitivity of the DNA-OLA from frozen peripheral blood mononuclear cells (PBMC) as compared to the OLA from virion RNA (plasma) and RNA consensus sequencing from dried blood spots.

3. To determine factors associated with virologic failure and drug resistance development.

4. To design a simple and integrative display of clinical/immunological progression of HIV disease after ART initiation.

## Methods

### Patient Population and Study Procedures

From 2002–2005, study participants had undergone standard medical procedures and routine HIV medical care at the Infectious Diseases Service at the INSN. According to the MINSA Guideline for the Management of the HIV-Infected Children, CD4+counts had been determined every 3 months, and viral load every 6 months at the Peruvian National Institutes of Health (Instituto Nacional del Salud, INS) [16]. Antiretroviral therapy for eligible patients was provided free of charge by the MINSA. Eligibility criteria for ART provided by the Peruvian Ministry of Health included: Established perinatal HIV infection^a^ and age < 18 months, or age >18 months and CDC immune category 2 or 3. Exceptions were planned for asymptomatic patients with a rapid decline in CD4+ or viral load >100,000cp/ml (or >10,000-20,000 in those > 30 months) [16]. Ethics approval was obtained by the respective institutional review boards (IRB) in the US and Peru.

For the cross-sectional analysis in 2005, all eligible subjects undergoing ART according to the MINSA program who agreed to participate and whose parents/guardians had signed the informed consent, were included. Basic clinical and virologic parameters from the start of ART in the individual patient until the date of testing were extracted from routine medical records and laboratory reports (viral load and CD4 testing data). Additional parameters were obtained, such as CDC stage [27], opportunistic and other infections, medication and dosing information, and adverse events attributable to ART. A previously published standardized adherence questionnaire (*PACTG P1042S*) was used at the time of cross-sectional analysis to systematically measure adherence based on information provided by parents and caregivers [28,29].

At the time of the first regular follow-up visit after entry into the study, routine blood sampling was again performed at the INS. In addition, 5 ml of citrated blood were collected from study participants for resistance testing. In addition, two Guthrie filter cards were collected with 4 capillary blood spots (finger prick) of 50 uL each.

### Virologic testing

Ficoll-Hypaque centrifugation and separation of the citrated blood was performed at the PRISMA laboratory in Lima. Plasma and PBMC were immediately stored separately at -20C and shipped on dry ice to the Tulane and LSU PACTU laboratory for RNA and DNA extraction. Viral loads in plasma were quantified by real-time RT-PCR as described [30].

The OLA was conducted according to the NIH protocol for mutations at HIV-1B protease positions D30N, I50V, V82A, V82S, V82T, I84V, N88D, and L90M as well as reverse transcriptase positions K103N, Y181C, K65R, T215F, T215Y, M184V, and Q151M [21,31]. Dried Blood Spots (DBS) collected on Guthrie cards were stored at room temperature to be shipped to the Stanford Center for AIDS Research for consensus RNA sequencing [32].

### Definition of virologic failure

For the purposes of the study, virologic failure was defined by two or more consecutive HIV RNA measurements above the detection limit (400cp/ml), 4 – 6 months after the initiation of ART therapy in patients where ≥ 2 viral load measurements were available. In patients P016T, P021T, P041T, P053T and P057T only two viral load measurements were available in total. These patients all showed signs of virologic failure indicated by HIV RNA measurements > 400cp/ml > 10 months after treatment initiation.

### Sample size calculation

We assessed the population size *N* needed for assessing differences in resistance development between patients failing ART and those successfully treated.

We assumed that 50% of patients would eventually fail ART *P* (failure) = 0.5 and that those failing ART would with 90% probability develop drug resistance *P* (res. |failure) = 0.9.

Conversely, successfully treated patients may with 10% probability develop resistance *P *(res.|sucess) = 0.1. We can therefore compute the expected number of patients with failure and resistance a = *P*(res. |failure)●*P*(failure)●*N*, with failure and no resistance *b* = (1-*P*(res. |failure))●*P*(failure)●*N*, with no failure and resistance *c* = *P*(res. |success)●(1-*P *(failure))●*N* and with no failure and no resistance *d* = (1-*P*(res. |success))●1-*P*(failure))●*N*. According to Fisher’s exact statistics $p=\frac{\left(\begin{array}{c} a+b\\ a \end{array}\right)\left(\begin{array}{c} c+d\\ c \end{array}\right)}{\left(\begin{array}{c} N\\ a+c \end{array}\right)}$ for the underlying contingency table, we could show significance at the 5% level (*p* ≤ 0.05) for a sample size of *N* = 12. For values *P*(res. |failure) = 0.8, *P*(res. |failure) =0.7 and *P*(res. |failure) = (1-*P*(res. |sucess)) population sizes of *N* = 12 and *N* = 22 would be required.

### Rates of clinical/immunological progression

For the purpose of this analysis, CDC categories were applied in a novel way, assigning new CDC categories at each assessment time point ignoring previous CDC scores.

The rates of clinical and immunological progression *r*_C_ and *r*_*I*_ respectively (average change of CDC score per year throughout the study population) were computed with the following formula

$\left(\begin{array}{c} r_{I}\\ r_{C} \end{array}\right)=\left(\begin{array}{c} \sum_{m_{I}}F_{m,I}\cdot m_{I}\\ \sum_{m_{C}}F_{m,C}\cdot m_{C} \end{array}\right)$, where *m*_*I*_ and *m*_*C*_ denote the magnitude (number of scores) of change observed and *F*_*m,I*_ and *F*_*m,C*_ the fractions that have changed by that magnitude within a certain time interval. For our evaluation, we computed the rates of immunological and clinical progressionfrom enrolment throughout years 1, 2 and and beyond (>=3).

### Assessment of the nutritional status using standard scores (Z-scores)

Malnutrition in the study population was assessed in terms of standard scores (z-scores) of child weight at enrolment in relation to the WHO reference weight [33]. The standard scores are defined by $z=\frac{x-\mu }{\sigma }$, where *x* represents the child’s weight and μ and σ denote the average weight within the child’s age category based on the WHO reference and standard deviation, respectively [33]. A standard score of z = −2 therefore denotes that the child’s weight is two standard deviations below average (i.e. *x* = μ-2σ).

## Results

### Demographics

A total number of 46 children were enrolled between September 2002 and March 2005. Median age at enrolment was 5.6 years (range: 0.7-14y). The median viral load at enrolment was 1.7∙10^5^ RNA/ml (range: 2.1∙10^3^ – 1.2∙10^6^) and the median CD4-count was 232 cells/μL (range: 1–1591). Notably, five children had CD4 counts below 10 cells/μL. The median weight at enrolment was 18 kg (range: 5.5-45). Notably, 43/46 (93%) had negative z-scores for child weight compared to the WHO reference corresponding age group [33], indicating evidence of malnutrition in this cohort. The median z-score was −2 (range: -4 to 0). CDC clinical categories (according to the 1994 Revised Classification System for HIV Infection in Children [27]) were attributed to each patient at baseline and again with each follow-up visit. Seven children were classified as clinical category N (not symptomatic), 4 children fell into clinical category A (mildly symptomatic), 15 were in category B (moderately symptomatic) and 20 were in category C (severely symptomatic). Notably, eight children (17%) were co-infected with active tuberculosis at enrolment. Children were also staged with respect to immune categories, according to the 1994 CDC classification system [27]. Four children were in category 1, 11 were in category 2, and 31 fell into category 3. Basic demographic characteristics are displayed in Table 1.

**Table 1:** Basic Characteristics of Study Participants

	**All**	**With subseq. virol. failure**	**Without subseq. virol. failure**
	n = 46	n = 26	n = 20
Gender (male n)	27	16	11
Age (years)	5.6 (0.2;14)	5.0 (0.67; 13.9)	6.5 (0.7; 13.8)
Weight below WHO child reference (n) [33]	43	24	19
Weight median z-score (range)	−2.0 (−4; 0)	−2.5 (−4; 0)	−1 (−4; 1)
Baseline viral load (RNA/ml)	1.7e5 (2.1e3;1.2e6)	2.1e5 (2.4e4; 1.1e6)	8.4e5 (2.1e3; 1.2e6)
CD4 count (cells/μL)	232 (1; 1519)	154 (1; 1591)	381 (2; 870)
Tubercoulosis coinfection (n)	8	3	5
**Clinical CDC stage**			
N (not symptomatic)	7	5	2
A (mildly symptomatic)	4	3	1
B (moderately symptomatic)	15	7	8
C (severely symptomatic)	20	11	9
**Immunological CDC stage**			
1	4	1	3
2	11	1	10
3	31	24	7

Table 1: Demographic characteristics and baseline disease status of study participants.

Vertical HIV transmission was the mode of infection for all but two children, who were infected by blood transfusion. Seven mothers had received antiretroviral prophylaxis with AZT +/− NVP for the prevention of mother-to-child transmission (pMTCT). Three children had been exposed to postnatal AZT for pMTCT (P019T, P020T, P028T). Four children had been exposed to unsupervised ART prior to enrolment: two children (P057T, P067T) received 3TC+AZT prior to enrolment. One child (P067T) continued NFV+3TC+AZT without any gap, while P053T and P016T had received NFV+3TC+AZT prior to initiation of the program. One child P016T continued with only a few weeks interruption, whereas for P053T there was a gap of one year between his prior ART medication and ART medication provided through this program. Throughout the study period, standard treatment was modified in five children (P007T, P011T, P019T, P031T and P057T). In these children, one component of their ART regimen was substituted respectively: AZT was replaced by stavudine (d4T) in P011T and P031T, 3TC was replaced by didanosine (DDI) in P057T, and NFV was replaced by nevirapine (NVP) in P007T and P019T.

### Viral dynamics and virologic failure rates

**Figure 1:**
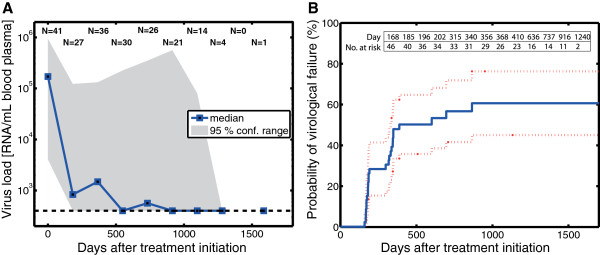
**Viral Load Dynamics and Probability of Virologic Failure. A:** Central tendency of the viral load dynamics after treatment initiation. The solid blue squares indicate the median viral load for all patients together with the confidence range spanned by the 5th and 95th percentiles (grey shading). The numbers at the top of the figure, e.g. N = 30, indicate the number of patients that gave rise to the estimates of the median viral load and its confidence area for the respective time points. **B:** Kaplan-Meier estimate of the cumulative probability of virologic failure after treatment initiation.

The central tendency of viral dynamics is shown in Figure 1A. The corresponding viral load measurements for all children are displayed in Additional File 1. Virologic failure was defined by two or more measurements demonstrating > 400 copies/ml RNA after 16 weeks of treatment (see filled squares in Additional file 1). The cumulative probability of virologic failure is shown in Figure 1B.

As can be seen, 44% of children experienced virologic failure during the first year of ART, half of the children failed before the end of the second year of ART. By the end of the study, 60 ± 16% had experienced virologic failure.

Both patients who had been infected by blood transfusion (2/2) and all children with previous ART exposure (4/4) eventually experienced viral failure. None of the 7 children whose mothers had received pMTCT prophylaxis with AZT +/− NVP (0/7) and none of the children who had received post-natal AZT prophylaxis for pMTCT (0/3) experienced virologic failure.

Children who were younger at entry were slightly more likely to fail ART (p = 0.06 by Wilcoxon rank sum test). Virologic failure was significantly associated with the immunologic CDC-score at baseline (i.e. when starting structured ART; p < 0.001 by cross-tab χ^2^ test), with severely immunosuppressed patients being most likely to fail ART.

In contrast, the CDC clinical category at baseline was not predictive of virologic failure during subsequent ART. Children who had reported missing > 50% of doses (according to the adherence questionnaire administered) were also more likely to experience virologic failure (p = 0.05; cross-tab χ^2^ test).

### Rates of immunologic & clinical progression and child growth

Neither immunologic CDC classification, nor clinical CDC classification at enrolment were correlated with the age of the children (but with the time between infection and start of therapy, p = 0.39 and p = 0.83; test for non-zero correlation).

**Figure 2:**
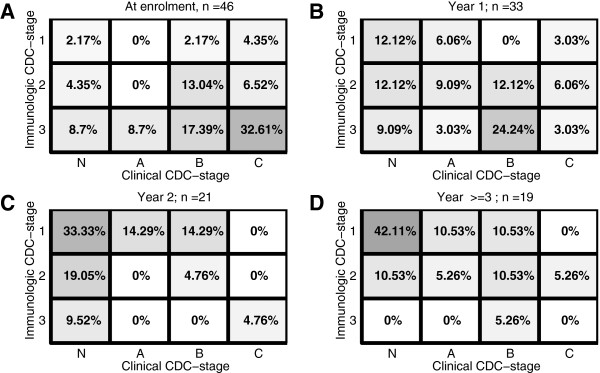
**Classification of Study Participants.** Immunologic and clinical classification of study participants at treatment initiation, throughout years 1 and 2, and ≥ 3 years after ART initiation. The numbers in the distinct fields and the intensity of the shading represent the percentage of individuals falling within the respective CDC classification. **A:** Classification at enrolment. **B:** Classification during year 1 after treatment initiation. **C:** Classification during year 2 after treatment initiation and **D:** Classification after year 2.

Study participants were classified in terms of CDC clinical and immune categories at enrolment, during year 1, during year 2, and after year 2, as shown in Figures 2A-D.

It can be seen in Figure 2A that at the time of enrolment, that the majority of study participants are clustered in the lower right corner (intensity of shading & percentages shown in the respective fields), which represents immunologic suppression (high immunologic CDC scores) and numerous opportunistic infections (immunologic scores ‘B’ & ‘C’). During year 1 after the onset of treatment (Figure 2B) the study participants’ scores are distributed almost equally throughout the space defined by the respective CDC clinical and immunologic classifiers. During year 2 after treatment initiation, most of the study participants showed evidence of immunologic recovery and an overall decrease in the number of clinical signs of HIV/AIDS, such as opportunistic infections (increasing percentages are found in the upper left corner in Figure 2C). After year two, a higher percentage of subjects are represented in the upper left corner of Figure 2D, while at the same time there is a slight regression to the right, indicating an overall clinical deterioration.

**Figure 3:**
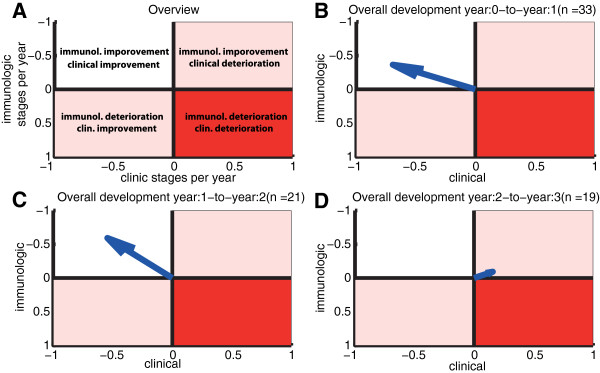
**Disease Progression.** Average rates of progression with respect to clinical and immune classifiers. **A:** The upper-left area indicates an overall improvement in terms of clinical and immune classifiers, whereas the upper-right area indicates immunological improvement but clinical deterioration. The lower-left area indicates immunological deterioration but clinical improvement, and the lower right area indicates deterioration with respect to both immunologic and clinical classifiers. **B:** The blue arrow indicates the overall rate of progression in the first year after treatment initiation (i.e. both clinical and immunologic parameters are improving). It was computed using the formula depicted in the Methods section (“Rates of clinical/immunological progression”). **C:** Overall rate of progression during the second year. **D:** Overall progression during the third year.

The overall rate of clinical/immunologic disease progression per treatment year is shown in Figures 3B-D: for the first year after enrolment (panel B), for the second year after enrolment (panel C), and for the time thereafter (panel D). It can be seen that antiviral treatment had a very positive effect on both immunologic and clinical parameters during the first year after ART initiation as well as during the subsequent year (the blue arrow pointing towards the upper-left in Figures 3B and C). The rate of improvement was −0.4 immunologic stages and −0.77 clinical stages during the first year after treatment initiation and −0.65 immunologic and −0.61 clinical stages from year 1 to year 2.

Immunologic improvement was minimal during year three (−0.1 stages), whereas the clinical status of the study participants worsened slightly by 0.16 stages on average (the blue arrow pointing towards the upper-right in Figure 3D). The overall changes during year three are very small. Whether these minor changes are also observable in larger cohorts, or whether they indicate a stabilization of immunologic and clinical progression warrants further investigation.

The immunologic CDC-scores at the time of final assessment were significantly correlated with virologic failure (p<0.01; cross-tab χ^2^ test), with patients failing therapy showing higher scores (i.e. being more severely compromised immunologically), while the final clinical CDC-scores were not linked.

In summary, immunologic improvement became evident soon after initiation of ART and could be maintained in this cohort of first-line ART recipients, whereas the clinical improvement (with respect to CDC scores) seemed to lag behind, possibly due to the fairly advanced disease stages at baseline.

The median weight after 1, 2 and 3 years of treatment was 20 kg, 22.3 kg and 23 kg, respectively. The median z-score was −1. During the first year of ART, 72% of the children showed negative z-scores, 75% in year 2 and 67% in/after year 3, which is a considerable improvement over child weight at enrolment, 93% showed negative z-scores.

### Drug resistance testing

On average, drug resistance testing was performed at 2.4 years after the initiation of structured ART. Prior to the cross-sectional analysis of this treatment cohort, drug resistance information had not been available to direct the choice of treatment regimens. In ART-failing patients, the vast majority of drug resistance tests (96%) were performed at time points after virologic failure.

Samples for RNA consensus sequencing were transported as dried blood spots on Guthrie cards. RNA amplification for consensus genotyping was possible in 14/46 samples (including 3 samples with a viral load slightly below 400 cp/ml), in 4 instances only the protease gene (PR) could be sequenced. All RNA consensus sequencing data is provided in Additional file 2. Overall, 70% of HIV-1 RNA sequences were derived from individuals eventually failing ART. In the remaining cases, RNA could be amplified from two patients whose viral load had just dropped below 400cp/ml, one had repeated measurements slightly below the threshold.

Samples for DNA and RNA OLA testing were transported as frozen plasma and PBMC samples after Ficoll-Hypaque centrifugation and separation. Of these, RNA-OLA testing was performed successfully in 20/46 (43%), in one case only the protease mutations could be tested by RNA-OLA. All OLA data is provided in Additional File 3. As expected, the majority of samples yielding RNA-OLA results (80%) were derived from patients with detectable viral loads. DNA-OLA testing however was successful in almost all patient samples (45/46, 98%), of which 47% showed no evidence of virologic failure at the time of testing. Hence, DNA-OLA from frozen PBMC provided a sensitive tool for the cross-sectional assessment of archived drug resistance in this patient cohort. RNA consensus genotyping from dot blots and RNA-OLA from plasma virions yielded results predominantly in individuals with already established virologic failure (over-representing those with viral loads above the 400cp/ml threshold).

### Drug resistance mutations

The M184V reverse transcriptase mutation was detected in 80% of the sequenced RNA samples and tested positive in 74% and 47% by RNA-OLA and DNA-OLA, whereas thymidine associated mutations (TAMs: M41L, D67N, K70R, L210W, T215F/Y, K219Q/E [34]) were detected in 50% of sequenced viral RNA. Using RNA-OLA and DNA-OLA, the T215Y and T215F mutations tested positive in 47% and 42%, respectively.

The protease mutation D30N was detected in 43% of RNA genotyping samples and in 0% and 2% of available RNA- and DNA-OLA samples. The N88D and L90M protease mutations were detected in 36% and 21% of genotyping samples, in 25% and 20% of RNA-OLA samples, and in 42% and 44% of DNA-OLAs, respectively.

Children who were previously exposed to short-term antivirals showed significantly higher numbers of resistance mutations detected by DNA-OLA (p = 0.01 by Wilcoxon rank sum (WRS) test), but not by RNA-OLA (p = 0.26; WRS test) or genotyping (p = 0.18; WRS test) at the time of cross-sectional analysis. Virologic failure was strongly associated with the number of resistance mutations detected by DNA-OLA (p < 0.001; WRS test).

The detection of the M184V reverse transcriptase mutation (indicating 3TC resistance) by any of the three methods (genotyping, RNA-OLA or DNA-OLA) was significantly more frequent in patients with virologic failure (p = 0.07^b^, p < 0.05^c^ and p < 0.001^c^). Also, the mutations N88D and L90M (NFV resistance) were more frequently detected by DNA-OLA in patients with virologic failure (p < 0.001 and p < 0.05, respectively; WRS test). The protease mutation D30N was not detected more commonly in cases of virologic failure (by any of the assays used), neither were TAMs selected differentially in failing vs. non-failing patients.

Detection of the M184V, N88D and L90M substitutions by RNA OLA was highly sensitive for virologic failure (sensitivity: 0.93, 1.0 and 1.0; binary classification test). The ability to obtain positive results with the RNA OLA, along with the detection of mutations M184V, N88D and L90M, may thus suggest virologic failure in this cohort of patients.

The detection of the same mutations (M184V, N88D and L90M) by DNA-OLA yielded a slightly lower sensitivity of 0.86, 0.9 and 0.75 for virologic failure, but the assay could be performed in almost all patient samples (regardless of virologic success or failure) indicating that virologic failure may indeed be attributed to resistance development at these three residues (these specific mutations appear significantly more frequently in failing patients, see Table 2).

**Table 2:** Frequency of Mutations Detected by Different Assays

	**M184V**	**TAM**	**n**	**Virol. Failure**	**D30N**	**N88D**	**L90M**	**n**	**Virol. Failure**
**RNA Genotyping**	**80**%^*^	50%	10	70%	43%	36%	21%	14	70%
**RNA-OLA**	**74**%^**^	47%^3^	19	84%	0%	25%	20%	20	80%
**DNA-OLA**	**47**%^***^	42%^3^	45	53%	2%	**42**%^***^	**44**%^**^	45	53%

Table 2: Frequency of mutations detected by RNA genotyping, RNA-OLA and DNA-OLA.

^*^associated with virologic failure (p < 0.1),

^**^strongly associated with virologic failure (p < 0.05),

^***^ very strong association with virologic failure (p < 0.001).

^3^ only T215F and T215Y.

### Relative sensitivities and specificities of the DNA- and RNA-OLA

We evaluated the DNA-OLA and RNA-OLA relative to each other in terms of a binary classification test: The DNA-OLA yielded a sensitivity of 59% relative to the RNA-OLA. Its relative specificity was 96%. Reversely, the sensitivity of the RNA-OLA relative to the DNA-OLA was 86%, whereas its specificity was 88%. (Table 3)

**Table 3:** Detection of Resistance Mutations with DNA-OLA vs. RNA-OLA

	**DNA+**	**DNA-**	**Sum**
RNA+	36	25	61
RNA-	6	278	184
Sum	42	203	

Table 3: Comparison of DNA-OLA and RNA-OLA. The field ‘DNA+/RNA+’ denotes the number of resistance mutations positively detected by both DNA-OLA and RNA-OLA, whereas the field ‘DNA-/RNA+’ denotes the number of resistance mutations where the DNA-OLA yielded a negative result and the RNA-OLA yielded a positive result.

## Discussion

There are two important aspects in this patient cohort, characteristic of ART cohorts in resource-limited settings: a) all patients received the same first-line antiretroviral regimen and b) patients, on average, were in advanced stages of HIV/AIDS when starting their first antiretroviral regimen [35]. When antiretroviral therapy was first introduced in Peru, uniform criteria were established by the MINSA to ensure the allocation of resources and medication to those most in need. This first cohort of patients at the largest children’s hospital in Peru suddenly became eligible for therapy at a time when many had already progressed to disease stages beyond the eligibility threshold.

The effect of delayed access to ART in this first cohort becomes evident in comparison to a recent study observing the transmission of resistant virus in a much younger cohort of neonates and children with timely access to pMTCT and ART in Peru, revealing a predominance of NNRTI mutations, whereas mutations conferring high-level resistance to ARV were still found to be rare [18]. This observation is unlikely an effect of age. Even though our cohort started treatment after the disease had progressed significantly, age by itself was not associated with an advanced clinical stage at enrollment. To the contrary, young age (thus earlier treatment initiation) seemed to favor virologic failure. This may also be due to a survivor effect, i.e. slower progression in those patients who had already survived the first years after MTCT.

Chances of virologic failure were high in this first pediatric cohort gaining access to ART in Peru in 2002/3, with ~44% showing virologic failure after the first year of ART, ~53% after two years. The majority of children were in poor health, as evidenced by malnutrition 93% of children below the reference weight for the respective age group [33]) and a high prevalence of opportunistic infections. Of note, 43% showed AIDS-defining conditions and 17% co-infections with active tuberculosis. Immunologically, 67% of the children had already r.eached the immunologic CDC category 3 (corresponding to an adult CD4 levels of < 200 cells/μL) prior to gaining access to structured ART.

Immunologic classification at baseline was very predictive for virologic failure. In agreement with studies in industrialized countries [36,37], these findings indicate that the percentage of CD4 cells in children with HIV/AIDS (i.e. the immunologic category) could be used to guide treatment initiation. In fact, the immunologic classification may be more valuable for the decision of ART initiation than relying on DNA-PCR results alone [38].

Despite relatively high rates of virologic failure in this cohort, both immunological and clinical conditions improved during ART, in particular throughout the first and second years of treatment. Thereafter little additional improvement was achieved. Overall, from the time of initiation of ART up until the time of the cohort assessment, 57% had showed marked improvement with respect to their clinical status (as measured by CDC category/visit), whereas 35% were unchanged clinically, and only 8% showed disease progression. With respect to the immunologic CDC-scores, 76% had improved, 22% had experienced no change, and 2% showed a decline in CD4 counts.

For improved visualization of the overall development of treatment cohorts during ART, we summarized the clinical and immunological response to therapy in an innovative fashion using a Clinical Course Integrated Display (CCID) with 3-by-4 tables based on the revised CDC clinical and immunological categories [27]. Here, we applied the CDC scores as a flexible tool to examine the cohort on a yearly basis, allowing for CDC scores to improve or deteriorate, according to the CD4 counts and reported clinical symptoms. Using this simple system in cross-sectional analyses and surveillance programs, rates of disease progression (Figure 3) may be computed for different cohorts allowing the comparison of treatment strategies in terms of their clinical and immunologic effects in a given population. This system may be applicable to similar cohort studies in developed and developing countries alike, especially in conjunction with cross-sectional analyses of antiretroviral drug resistance.

Previous exposure to (often incomplete) ART was significantly associated with virologic failure, indicating that short courses of unsupervised ART prior to the initiation of coordinated long-term treatment programs may be counterproductive as they may lead to the rapid development of drug resistance. Archived drug resistance mutations, acquired during previous exposures to antiretrovirals and still present in the PBMC compartment may be detected reliably by DNA OLA.

Exposure of the newborn to post-natal pMTCT with AZT did not increase the likelihood of subsequent virologic failure, neither did maternal exposure to pMTCT with AZT +/− NVP. There are three possible explanations why pMTCT did not affect subsequent treatment success:

a) The pMTCT did not lead to a transmission/selection and “archivation” of drug resistance,

b) Although drug resistance against the pMTCT regimen (i.e. AZT +/− NVP) developed and was archived, it did not impede the success of subsequent triple-drug ART consisting of AZT + 3TC + NFV.

c) Drug resistance did not persist until the initiation of ART.

In fact, in only one child (P028T) we detected archived drug resistance by DNA OLA (mutation 215Y; AZT resistance) at the time of cross-sectional resistance testing. This child (P028T) did not encounter virologic failure (hinting towards scenario b).

Drug resistance in the context of pMTCT may emerge- or be transmitted - by two possible mechanisms:

(i) Drug resistant virus is selected in the mother and passed on to the child (e.g. during birth or breastfeeding).

(ii) The newborn is infected with susceptible virus and subsequently selects drug resistant virus, e.g. during ARV exposure.

Ad (i): When a single dose of antivital medication for maternal pMTCT is administered at the onset of labor, it is rather unlikely that drug resistant virus is passed on to the child. Although the pMTCT regimen may induce a selective pressure on the maternal virus, there is hardly enough time for this virus to be selected to sufficient numbers to be transmitted during birth, see also [39]. However, drug resistant virus may, with some probability, be transmitted during subsequent breastfeeding [39].

Newborns P019T, P020T and P028T were not breastfed and their mothers received a single dose of AZT +/− NVP shortly before birth. However, these newborns received 6 mg/day (P019T, P020T) or 28 mg/day (P028T) of post-natal AZT. As explained above, postnatal AZT administered to P028T may explain the archiving of AZT resistance in the child’s PBMC DNA (case ii). However, this did not lead to subsequent therapeutic failure (case b).

The mothers of newborns P002T, P003T, P027T and P046T were breastfeeding. They received extended AZT for periods shorter than the actual duration of breastfeeding. None of these children (P002T, P003T, P027T and P046T) showed evidence of archived AZT resistance based on DNA-OLA at the time of cross-sectional assessment. These children could have either been infected with susceptible virus during labour, or during breastfeeding (after cessation of extended maternal AZT), or else resistance may not have persisted until treatment initiation or until the DNA OLA was performed.

A potential weakness of a cross-sectional study design is that clinical and laboratory data from the beginning of ART up until the date of cross-sectional analysis had to be extracted from medical records and parent/patient interviews. Adherence data using the PACTG questionnaire are always self-reported. This study design does not allow for detailed cause-effect analyses, prospective surveillance and follow-up visits, or the assessment of mortality data. The cross-sectional analysis however does reflect the real-world effectiveness of a medical intervention in a low-resource setting, which often includes patients who would not typically be able to participate in controlled clinical trials. The focus of this study was the assessment of the usefulness of cross-sectional resistance testing using the DNA versus RNA OLA.

The DNA OLA may be particularly useful for the purposes of population-based surveillance in low resource settings where genotyping tests may not be readily available. The DNA-OLA was very indicative for the presence of resistance (high specificity, low false positive rate), but less indicative for the absence of resistance (low sensitivity, high false negative rate) in comparison to the RNA OLA. To the contrary, the RNA-OLA was more useful to determine the absence rather than the presence of drug resistance. Therefore, DNA-OLA can be used to rule-in resistance, whereas RNA-OLA may be used to rule-out resistance.

The detection of the resistance mutations M184V, N88D and L90M by DNA-OLA was highly sensitive for virologic failure in this cohort treated with lamivudine-azidothymidine-nelfinavir as first-line therapy. The analysis of archived HIV-DNA resistance in PBMC provided useful results in most patients, even if virologic failure was not (yet) evident. The DNA-OLA may detect resistance mutations that have been acquired during previous exposure to erratic short-term ART, still present in the lymphocyte compartment. This may occur in low-resource settings before antivirals become universally available, when patients and their families are restricted to temporary access to limited, often insufficient amounts of antiviral medications. Turnover rates within the lymphocyte compartment may however be too low for the early detection of antiretroviral drug resistance during therapy (i.e. in time before viral failure becomes apparent).

A possible strategy for the improvement of ART in resource-poor settings (where genotyping is often not available) could be to use the DNA-OLA as a baseline screening tool before starting therapy. This could be combined with the use of RNA-OLA in those patients experiencing virologic failure. Notably, a positive RNA OLA at positions M184V, N88D or L80M was highly sensitive for virologic failure (sensitivity: 0.93, 1.0 and 1.0, respectively). Therefore, drug resistance monitoring at key residues using RNA OLA in patients experiencing virologic failure may be particularly useful as an economical indicator of drug resistance and could suggest a treatment change.

Success rates could likely be improved even further if treatment was initiated at higher CD4 counts, in line with recent revisions of the treatment guidelines in industrialized countries (initiation of treatment at an adult CD4 count of 350 cell/μL) [36,37]. This is in agreement with recent reports from other cohorts in Latin America. A recent cross-sectional analysis and evaluation of clinical outcomes of ART in Latin America showed that nearly half of the patients were so-called “late testers/presenters”. Evaluations of outcomes with ART in Latin American children revealed a higher incidence of opportunistic infections when compared to US cohorts (such as PACTG 129C) [36,37].

While consensus RNA genotyping (if available) will likely remain the mainstay of individualized resistance testing during ongoing antiretroviral therapy, the applicability of the OLA in population-based surveillance remains to be fully assessed in larger cohorts, including cost-effectiveness analyses and assessments of the personnel and training required for either method. At the time of the study, genotyping was not available. In recent years, capacities for monitoring drug resistance have been expanded at the Peruvian INS including sequencing facilities and an e-health driven, web-based laboratory information system [40,41]. The national ART program was expanded in 2004 to include larger parts of the population living with HIV/AIDS, including infants in earlier stages of HIV infection [41-43].

Our data emphasize the need for timely antiretroviral treatment initiation and early HIV testing to contribute to this aim [5,12,35,44]. For children undergoing therapy, regular follow-up visits with viral load and resistance testing and concrete measures to monitor and improve adherence (using PDA’s, cellphone reminders and other e-health features) may be a key to success of ART in Latin America and beyond [45-52].

## Conclusions

1. HIV drug resistance was the major factor contributing to virologic failure of antiretroviral therapy in this cohort of children with delayed access to structured ART in Lima, Peru.

2. In most instances, virologic failure occurred early in the course of treatment and commonly after previous exposure to unsupervised ART, but not in relation to pMTCT.

3. The DNA OLA method detected antiretroviral resistance at key positions independently of virologic failure in the form of integrated DNA (in PBMC), whereas the RNA OLA detected antiviral resistance in viral RNA (in plasma) only after virologic failure. Antiviral resistance was more readily detected by OLA than by RNA consensus genotyping (from dried blood spots).

4. The DNA-OLA could be used prior to treatment initiation to rule-out archived drug resistance to standard regimens, in particular when previous exposure to antiretrovirals is anticipated. The RNA-OLA could be used to guide the choice of second-line antiretrovirals in patients switching ART regimens after experiencing virologic failure.

### Endnotes

^a^ confirmed by DNA-PCR/viral load at 6 months, or by ELISA at/after 18 months or AIDS-defining diagnosis

^b^ Fisher’s exact test

^c^ χ^2^ test

## Abbreviations

INS: Instituto Nacional del Salud (Peruvian National Institutes of Health); IESN: Instituto Especializado de Salud del Niño; PRISMA: Asociación Benéfica Proyectos en Informática, Salud, Medicina y Agricultura; MINSA: Ministerio de Salud del Peru; PACTG: Pediatric AIDS Clinical Trials Group; ART: Antiretroviral Therapy; MTCT: Mother-to-child transmission; pMTCT: Prevention of mother-to-child transmission; AZT: Azidotymidine; 3TC: Lamivudine (LMV); NFV: Nelfinavir; NRTI: Nucleoside-analogue Reverse Transcriptase Inhibitors; NNRTI: Non-nucleoside-analogue Reverse Transcriptase Inhibitors; PI: Protease Inhibitor; OLA: Oligonucleotide Ligation Assay; PCR: Polymerase Chain Reaction; RNA: Ribonucleic Acid; DNA: Desoxyribonucleic Acid; WHO: World Health Organization; HIV: Human immunodeficiency virus; AIDS: Acquired Immunodeficiency Syndrome.

## Competing interest

All authors declare no competing interests.

## Authors’ contributions

Study concept and design: BAR, RAO, RVD, DKK. Acquisition of data: BAR, GSC, MEC, LK. Laboratory Analyses: BAR, PC; AMA, JER, DKK. Analysis and interpretation of data: MVK, BAR. Drafting of the manuscript: BAR, MVK. Critical revision of the manuscript for intellectual content: DKK, RAO, RVD, AMA, GSC, PC. Statistical analysis: MVK. All authors read and approved the manuscript.

## Pre-publication history

The pre-publication history for this paper can be accessed here:

http://www.biomedcentral.com/1471-2334/13/1/prepub

## Supplementary Material

Additional file 1 Individual viral load dynamics in children after treatment initiation, stratified by responders (black solid dots) and children who experienced virologic failure (red squares). Click here for file

Additional file 2 **Sequencing Data.** Table with the raw viral sequencing data from dried blood spots. Click here for file

Additional file 3 **OLA Data.** Table with the raw OLA data from plasma (RNA-OLA) and PBMCs (DNA-OLA). Click here for file
