# Supplementary material for: Antiviral Resistance and Correlates of Virologic Failure in the first Cohort of HIV-Infected Children Gaining Access to Structured Antiretroviral Therapy in Lima, Peru: A Cross-Sectional Analysis
Source: BMC Infect Dis. 2013 Jan 2;13:1. doi: 10.1186/1471-2334-13-1 (PMC3782360; doi:10.1186/1471-2334-13-1)
Supplement: Additional file 1 — Individual viral load dynamics in children after treatment initiation, stratified by responders (black solid dots) and children who experienced virologic failure (red squares). [file 1471-2334-13-1-S1.pdf]

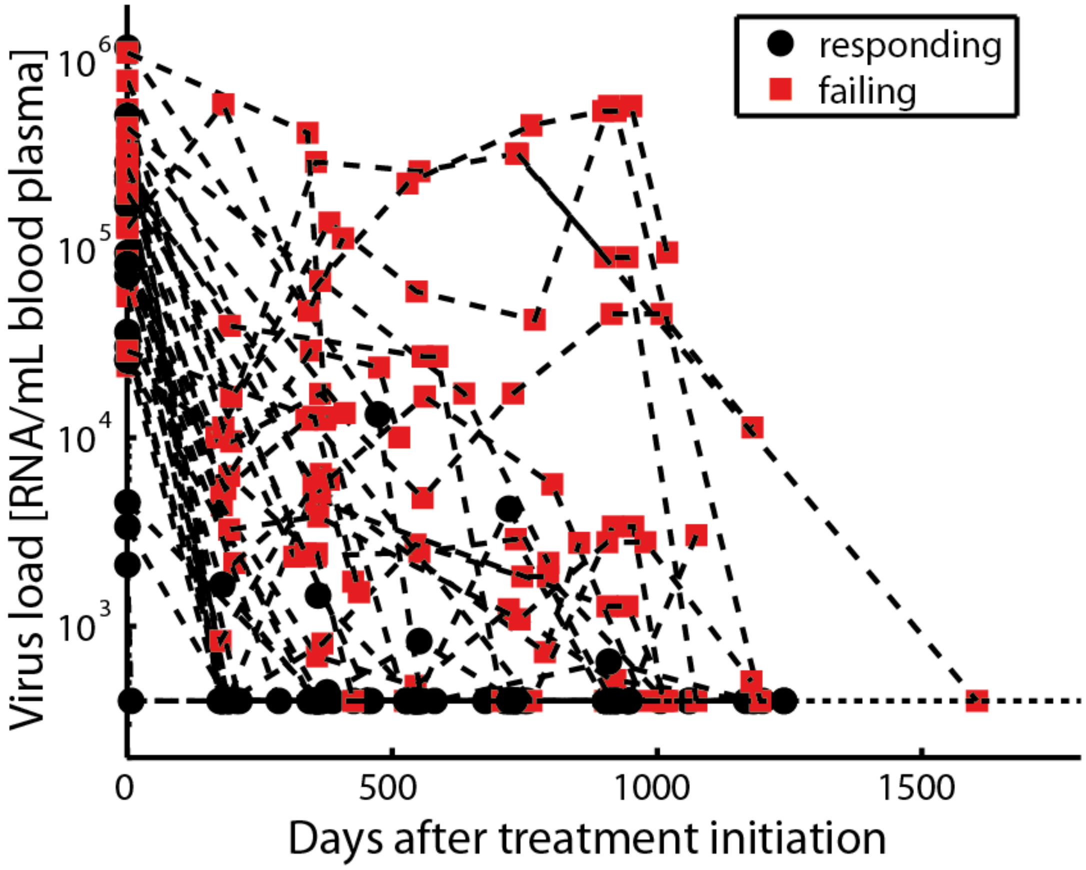

Additional Figure 1: Individual viral load dynamics in children after treatment initiation, stratified by responders (black solid dots) and children who experienced virologic failure (red squares).
